# Experimental co-infection of calves with SARS-CoV-2 Delta and Omicron variants of concern

**DOI:** 10.1080/22221751.2023.2281356

**Published:** 2023-11-08

**Authors:** Konner Cool, Natasha N. Gaudreault, Jessie D. Trujillo, Igor Morozov, Chester D. McDowell, Dashzeveg Bold, Taeyong Kwon, Velmurugan Balaraman, Patricia Assato, Daniel W. Madden, Emily Mantlo, Jayme Souza-Neto, Franco Matias-Ferreyra, Jaime Retallick, Gagandeep Singh, Michael Schotsaert, Mariano Carossino, Udeni B. R. Balasuriya, William C. Wilson, Roman M. Pogranichniy, Adolfo García-Sastre, Juergen A. Richt

**Affiliations:** a Department of Diagnostic Medicine/Pathobiology, College of Veterinary Medicine, Kansas State University, Manhattan, KS, USA; b Louisiana Animal Disease Diagnostic Laboratory and Department of Pathobiological Sciences, School of Veterinary Medicine, Louisiana State University, Baton Rouge, LA, USA; c Foreign Arthropod-Borne Animal Diseases Research Unit, National Bio and Agro-Defense Facility, United States Department of Agriculture, Manhattan, KS, USA; d Department of Microbiology, Icahn School of Medicine at Mount Sinai, New York, NY, USA; e Global Health and Emerging Pathogens Institute, Icahn School of Medicine at Mount Sinai, New York, NY, USA; f Department of Medicine, Division of Infectious Diseases, Icahn School of Medicine at Mount Sinai, New York, NY, USA; g The Tisch Cancer Institute, Icahn School of Medicine at Mount Sinai, New York, NY, USA; h Department of Pathology, Molecular and Cell-Based Medicine, Icahn School of Medicine at Mount Sinai, New York, NY, USA

**Keywords:** SARS-CoV-2, COVID-19, variants of concern, delta, omicron, cattle, experimental Infection

## Abstract

Since emerging in late 2019, severe acute respiratory syndrome coronavirus 2 (SARS-CoV-2) has repeatedly crossed the species barrier with natural infections reported in various domestic and wild animal species. The emergence and global spread of SARS-CoV-2 variants of concern (VOCs) has expanded the range of susceptible host species. Previous experimental infection studies in cattle using Wuhan-like SARS-CoV-2 isolates suggested that cattle were not likely amplifying hosts for SARS-CoV-2. However, SARS-CoV-2 sero- and RNA-positive cattle have since been identified in Europe, India, and Africa. Here, we investigated the susceptibility and transmission of the Delta and Omicron SARS-CoV-2 VOCs in cattle. Eight Holstein calves were co-infected orally and intranasally with a mixed inoculum of SARS-CoV-2 VOCs Delta and Omicron BA.2. Twenty-four hours post-challenge, two sentinel calves were introduced to evaluate virus transmission. The co-infection resulted in a high proportion of calves shedding SARS-CoV-2 RNA at 1- and 2-days post-challenge (DPC). Extensive tissue distribution of SARS-CoV-2 RNA was observed at 3 and 7 DPC and infectious virus was recovered from two calves at 3 DPC. Next-generation sequencing revealed that only the SARS-CoV-2 Delta variant was detected in clinical samples and tissues. Similar to previous experimental infection studies in cattle, we observed only limited seroconversion and no clear evidence of transmission to sentinel calves. Together, our findings suggest that cattle are more permissive to infection with SARS-CoV-2 Delta than Omicron BA.2 and Wuhan-like isolates but, in the absence of horizontal transmission, are not likely to be reservoir hosts for currently circulating SARS-CoV-2 variants.

## Introduction

Severe acute respiratory syndrome coronavirus 2 (SARS-CoV-2) has been circulating in the human population since its emergence in 2019. SARS-CoV-2 has repeatedly spilled back from humans into animals, occasionally establishing sustained transmission in felids, hamsters, mustelids, and white-tailed deer, and developing host-adapted mutations [1–6]. Epidemiological investigations of these outbreaks have revealed subsequent secondary spillover events where animal-adapted variants were found into humans. This has led to mass culling of mink and hamsters, resulting in significant economic losses and trade restrictions [7,8]. The threat of additional animal reservoirs being established has prompted increased surveillance efforts in companion, farmed, and wild animal populations. To date, nearly 700 outbreaks in 26 species have been reported by the WOAH [9].

Several *in silico* and *in vitro* methods have been applied to evaluate animal species for their susceptibility to SARS-CoV-2, however these predictions do not always translate when tested *in vivo* [10–16]. The emergence of SARS-CoV-2 variants of concern (VOCs) has made it necessary to re-evaluate the host–pathogen interaction, since many animal species have not been evaluated *in vivo* for their susceptibility to SARS-CoV-2 VOCs [17-19]. VOCs are characterized by mutations in the Spike (S) protein, particularly in the Receptor-Binding Domain (RBD), and are associated with enhanced transmissibility, changes in pathogenicity, evasion of pre-existing immunity from previous infections or vaccination, and/or decreased effectiveness of vaccines, therapies, and/or diagnostics [20,21]. Importantly, changes in host range have been described for SARS-CoV-2 VOCs, most notably in mice, that can be infected by alpha, beta, gamma and Omicron VOCs, but not by the ancestral or delta VOCs [18–20,22]. The expansion of host range for VOCs is largely attributed to key amino acid substitutions in the RBD of the S protein. These substitutions alter the binding affinity between SARS-CoV-2 Spike protein and the host ACE-2 protein, which serves as the cellular receptor for SARS-CoV-2.

Cattle (*Bos taurus*) have been evaluated for their susceptibility to Wuhan-like SARS-CoV-2 isolates using various methods, including *in silico* predictions, receptor-binding/affinity assays, replication kinetics in primary cell cultures and tissue explants, as well as by *in vivo* experimental infection [11,13,15,23–26]. Together, these data suggested that cattle were unlikely to support sufficient viral replication to contribute or sustain transmission of SARS-CoV-2 within cattle herds. However, seropositive cattle have since been identified in Italy and Germany, and SARS-CoV-2 RNA has been isolated from cattle in India and Nigeria, all coinciding with the Delta wave of SARS-CoV-2 transmission in humans, warranting further investigation into the susceptibility of this species to SARS-CoV-2 VOCs [27–30].

Here, we present our findings on the susceptibility and transmission of SARS-CoV-2 VOCs in calves after co-infection with the Delta and Omicron BA.2 VOCs. Our analysis includes clinical evaluations, viral RNA shedding and RNA distribution in respiratory and other tissues, virus isolation, pathological findings, and virus-specific antibody responses. Furthermore, RNA isolated from clinical and tissue samples was analyzed by next-generation sequencing to examine virus evolution and competition of these two VOCs *in vivo*.

## Materials and methods

### Ethics statement

All animal studies and experiments were approved and performed under the Kansas State University (KSU) Institutional Biosafety Committee (IBC, Protocol #1460) and the Institutional Animal Care and Use Committee (IACUC, Protocol #4508) in compliance with the Animal Welfare Act. All animal and laboratory work were performed in biosafety level-3 + and −3Ag laboratories and facilities in the Biosecurity Research Institute (BRI) at KSU in Manhattan, KS, USA.

### Cells and virus propagation

Vero E6 cells stably expressing transmembrane serine protease 2 (Vero-E6/TMPRSS2; [31]) obtained from Creative Biogene (Shirley, NY) via Kyeong-Ok Chang at KSU were used for SARS-CoV-2/Omicron BA.2 VOC propagation, titration, and isolation. Cells were cultured in Dulbecco’s Modified Eagle’s Medium (DMEM, Corning, New York, N.Y, USA), supplemented with 10% fetal bovine serum (FBS, R&D Systems, Minneapolis, MN, USA) and antibiotics/antimycotics (ThermoFisher Scientific, Waltham, MA, USA), and maintained at 37°C under 5% CO_2_ atmosphere. To maintain TMPRSS2 expression, a selection antibiotic, G418, was added to cell culture medium at 0.5 mg/mL but was not used during virus cultivation or virus assays. The SARS-CoV-2/Delta (hCoV-19/USA/NYMSHPSP-PV29995/2021; lineage B.1.617.2, clade GK) VOC is a clinical isolate provided by the Mount Sinai Pathogen Surveillance program (directed by Drs. van Bakel, Sordillo and Simon). Delta virus stock was propagated in a single passage on Calu3 cells. The SARS-CoV-2 Omicron BA.2 strain (Lineage B.1.1.529, BA.2; clade GRA) was acquired from BEI Resources (NR-56520; Manassas, VA, USA). Virus stock was produced by a single passage on Vero-E6/TMPRSS2 cells.

To determine infectious titers of virus stocks and inoculum, 10-fold serial dilutions were performed using Vero-E6/TMPRSS2 cells on 96-well cell culture plates. Cells were observed under a light microscope for the presence or absence of cytopathic effect (CPE) after at least 96 h of incubation at 37°C under 5% CO_2_ atmosphere. Tissue culture infective dose 50% (TCID_50_)/mL was calculated using the Spearman-Kaerber method [32].

### Virus challenge of animals

Ten male Holstein calves, approximately 4 months old, were purchased and transported from a South Dakota feedlot to KSU and held outdoors for an acclimation period of 2 weeks prior to challenge with SARS-CoV-2. Prior vaccinations included Nalsalgen, Virashield 6, Covexin 8, and Endovac; the animals also received Draxxin and Ivermectin. Upon arrival, the calves were examined by a KSU veterinarian. Several calves had enlarged prescapular lymph nodes (#673, 88, T7477), likely associated with recent vaccinations. Additionally, a mild cough was noted in calf #678 and mild respiratory signs in calf HT9.

**Figure 1.**
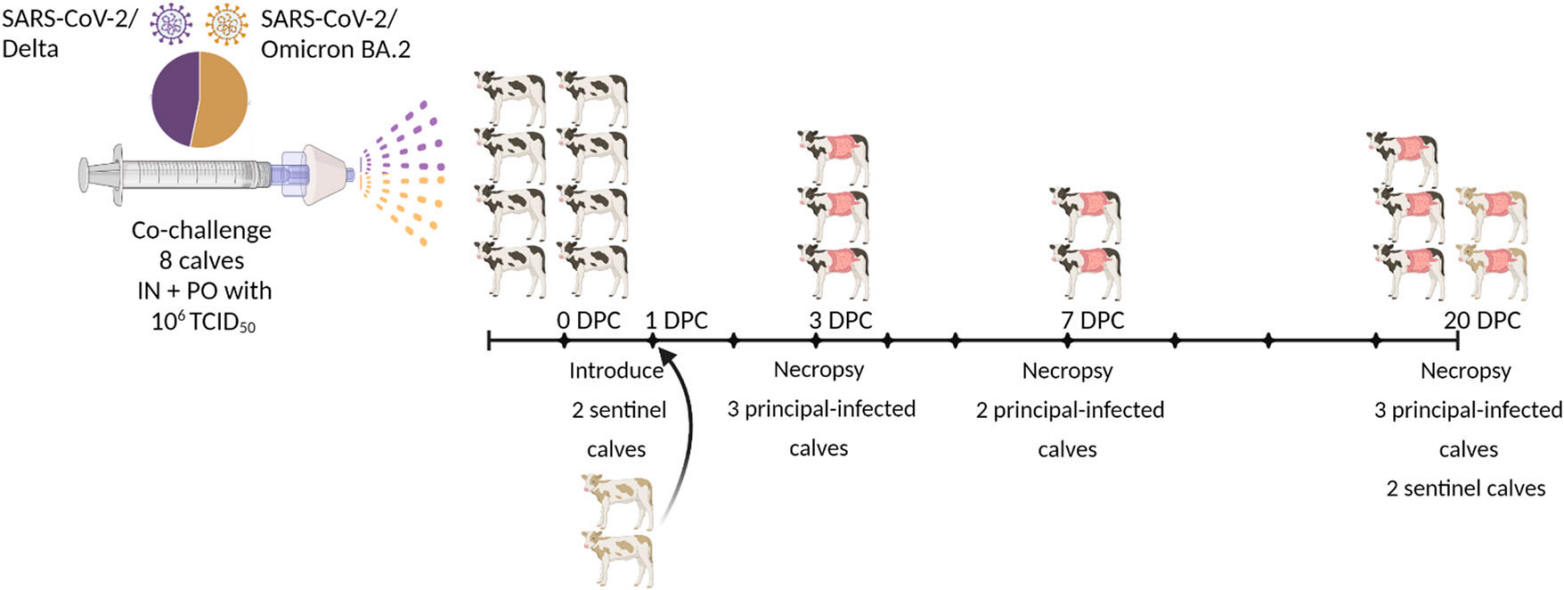
Experimental design. Eight male Holstein calves, approximately 4 months old, were administered a 50/50 mixture of SARS-CoV-2 Delta (B.1.617.2, clade GK) and Omicron BA.2 (lineage B.1.1.529; clade GRA) intranasally (IN) using a MAD Nasal™ atomization device (Teleflex, Morrisville, NC, USA), and orally (PO) by a micropipette, with a total dose of 1 × 10^6^ TCID_50_ in 4 mL (2.5 × 10^5^ TCID_50_/mL). Two sentinel calves were isolated in a separate pen, physically distant and up-current of directional airflow from the challenged animals. The sentinel calves were co-mingled with the eight principal-infected calves after sampling at 1 DPC, 24 h post-challenge. Whole blood (EDTA) was collected on −1, 3, 7, 10, 14, 18, and 20 days post-challenge (DPC). Serum was collected on −1, 3 (calves HT1, HT8, 678 only), 7, 10, 14, 18, and 20 DPC. Nasal, oral, and rectal swabs were collected on −1, 1 through 5, 7, 10, 14, 18, and 20 DPC. Calves were humanely euthanized and postmortem examinations were performed at 3 (n = 3 principal-infected), 7 (n = 2 principal-infected), and 20 DPC (n = 5; 3 principal-infected, and 2 sentinels).

After the acclimation period, all calves were transported to one large BSL-3Ag room at the BRI. A 50/50 mixture of SARS-CoV-2 Delta and Omicron BA.2 VOCs was administered to eight calves intranasally (IN), using a MAD Nasal™ atomization device (Teleflex, Morrisville, NC, USA), and orally (PO), administered with a micropipette, simultaneously for a total volume of 4 mL containing a total of 1 × 10^6^ TCID_50_ (2.5 × 10^5^ TCID_50_/mL; 0.5 × 10^6^ TCID_50_ for each VOC). The infected 8 calves are considered the principal-infected animals. Two sentinel calves were isolated in a separate pen, physically distant and up-current of directional airflow from the SARS-CoV-2 challenged animals. The sentinel calves were introduced to the eight principal-infected calves after sampling at 1 DPC, approximately 24 h post-challenge (Figure 1).

### Clinical evaluations and sample collection

Calves were observed daily and evaluated for clinical symptoms including respiratory distress, gastrointestinal irregularities, appetite, activity levels, and elevated body temperatures. Nasal, oral, and rectal swabs were collected on days −1, 1 through 5, 7, 10, 14, 18, and 20 post-challenge. Samples were collected with FLOQSwabs® (Copan Diagnostics, Murrieta, CA) in 1 mL DMEM supplemented with 1% antibiotics/antimycotics (ThermoFisher Scientific, Waltham, MA, USA), placed directly on ice, and transported to the BSL-3 + lab for processing. Whole blood (EDTA) was collected on days −1, 3, 7, 10, 14, 18, and 20 post-challenge. Serum was collected on days −1, 3 (# HT1, HT8, 678 only), 7, 10, 14, 18, and 20 post-challenge.

At 3 days post-challenge (3 DPC) three principal-infected calves were euthanized (HT1, HT8, 678) and full postmortem examinations were performed. Two additional principal-infected calves (# 673 and T7478) were euthanized, and postmortem examinations performed at 7 DPC. The remaining 5 calves (3 principal-infected and 2 sentinels) were euthanized, and postmortem examinations performed at the end of the study (20 DPC). During postmortem examination, the head including the entire upper respiratory tract and central nervous system (brain), trachea and lower respiratory tract, lymphatic and cardiovascular systems, gastrointestinal (GI) tract, urogenital system, and integument were grossly evaluated. Lungs were evaluated for gross alterations such as edema, congestion, discoloration, atelectasis, and consolidation/pneumonia. Cerebrospinal fluid (CSF) was collected by syringe and needle via the atlanto-occipital (C0-C1) joint, and bronchoalveolar lavage fluid (BALF), nasal wash and urine were collected and stored at −80°C until processed for virus isolation/RNA detection. Tissue samples from the respiratory tract, nasal turbinate (rostral and ethmoturbinates), trachea at multiple levels, all lung lobes, gastrointestinal tract and visceral organs (spleen, kidney, liver, heart), tonsils and lymph nodes (retropharyngeal, mandibular, tracheo-bronchial and mesenteric), brain including olfactory bulb, and bone marrow were collected and either fixed in 10% neutral-buffered formalin (Fisher Scientific, Waltham, MA, USA) for histopathologic examination or frozen for reverse transcriptase quantitative PCR (RT-qPCR) and virus isolation.

### RNA extraction and reverse transcriptase quantitative PCR (RT-qPCR)

To detect SARS-CoV-2 specific RNA, nucleic acids were extracted from swab samples, whole blood, and tissue homogenates using a magnetic bead based total nucleic acid extraction system and quantified using the N2 primer/probe set from the CDC COVID-19-novel coronavirus real-time RT–PCR diagnostic panel as described previously [33]. Briefly, swabs in DMEM or 20% weight:volume tissue homogenates in DMEM were combined in equal amounts with RNA stabilization/lysis Buffer RLT (Qiagen, Germantown, MD, USA) and vortexed. Sample lysates were transferred into Taco extraction plates (GeneReach, USA) loaded with GeneReach reagents and extracted following the manufacturer’s instructions with minor modifications as previously described [33]. Additionally, an exogenous internal positive control was added to the extraction to monitor extraction efficiency and consistency, and downstream inhibition of the RT-qPCR reaction [34].

Following extraction, duplicate RT-qPCR reactions were set up as follows: 5 µL of eluent was combined with qScript XLT One-Step RT-qPCR Tough Mix (Quanta BioSsciences, Beverly, MA, USA) and N2 targeted primers/probes (CDC COVID-19-novel coronavirus real-time RT–PCR diagnostic panel) and run as 20 µL reactions on a 96-well PCR plate (BioRad, Hercules, CA, USA). RT-qPCR reactions were carried out on a CFX96 Real-Time thermocycler (BioRad, Hercules, CA, USA) using a 20-minute reverse transcription step and a 45 cycle PCR. A quantitated PCR positive control (IDT, IA, USA; 2019-nCoV_N_Positive Control, diluted 1:100) and four non-template control (NTC) samples were included on every plate. A reference standard curve method using a 10-point standard curve of quantitated viral RNA (USA-WA1/2020 Wuhan-like isolate) was used to quantify RNA copy numbers. A positive Ct cut-off of 38 cycles was used. Data are presented as the mean of the calculated N gene copy number per mL of liquid sample or per gram of a 20% tissue homogenate.

### Next-generation sequencing

Virus stocks were sequenced by next-generation sequencing (NGS) using the Illumina NextSeq platform (Illumina, San Diego, CA, USA). The consensus sequence of the SARS-CoV-2 Delta variant (B.1.617.2) was found to have 100% sequence similarity to the reference sequence in GISAID (accession number: EPI_ISL_2290769). The Omicron BA.2 virus stock sequence was obtained by mapping reads to the reference sequence for SARS-CoV-2 Omicron BA.2 on GISAID (accession number: EPI_ISL_8643930), followed by extraction of the consensus sequence. One mutation in ORF1ab (NSP6) was present at 54% (A3694 V).

Data generated from NGS on the Illumina NextSeq platform (Illumina) was used in conjunction with the VirStrain [35] analysis package to determine the genetic composition (% lineage) of SARS-CoV-2 RNA present in virus stocks, clinical swab samples, and tissue homogenates. SARS-CoV-2 DNA amplicons were generated from viral RNA using the Midnight V.6 protocol (dx.doi.org/10.17504/protocols.io.bwyppfvn), a tiled primer amplification protocol. Library preparation of amplified SARS-CoV-2 DNA for sequencing was performed using a Nextera XT library prep kit (Illumina) following the manufacturer’s protocol. The libraries were sequenced on the Illumina NextSeq using 150 bp paired end reads with a mid-output kit. Reads were de-multiplexed and parsed into individual sample files that were imported into CLC Genomic Workbench version 21 (Qiagen) for analysis. Reads were trimmed to remove primer sequences and filtered to remove any short and low-quality reads (Q-score < 25). FASTQ files of the filtered and trimmed reads were exported for downstream analysis (Supplementary Table 3). Reads were then analyzed on VirStrain (Version 1.12) to determine SARS-CoV-2 lineage/strain. The genome sequences from the virus stocks used for animal challenge were used to construct the reference database for VirStrain lineage assignment used for this study. The VirStrain software was used to build a library of sample sequence SNPs based on the inoculum used as the reference sequences.

### Virus isolation

Samples taken from calves post-challenge with Ct values <30 were subjected to a single passage on Vero-E6/TMPRSS2 cells and subsequent indirect immunofluorescence assay (IFA) using SARS-CoV-2-specific monoclonal antibodies to confirm the absence/presence of infectious virus. Virus isolation was performed on 24-well cell culture plates seeded at a density of 1 × 10^5^ cells/well with low passage Vero-E6/TMPRSS2 cells 24 h prior to infection. Tissue homogenates were diluted 1:10 in DMEM, syringe filtered through a 0.22 µm filter, and 150 µL was added per well in 12 replicates. Diluted homogenates were incubated on cells for 1 h at 37°C under 5% CO_2_ atmosphere, then removed, washed with phosphate buffered saline (PBS), and replaced with 500 µL of fresh DMEM supplemented with 5% FBS and 1% antibiotics/antimycotics. Plates were then incubated as described above and observed daily for CPE. After 72 h, the media was removed, and wells were washed with 1x PBS and then fixed with cold 100% methanol and incubated at −20°C for 10 min. Plates were then washed three times with 1x PBS. A mixture of SARS-CoV-2 N- and RBD-specific monoclonal antibodies, produced in-house, were diluted 1:5 in 1x PBS + 1% BSA, added to the wells and incubated at room temperature for 1 h. Following three washes with 1x PBS + Tween-20 (0.05%), Alexa Flour™ 488 Goat anti-mouse IgG (ThermoFisher Scientific, Waltham, MA, USA) secondary antibody, diluted 1:1000 in 1x PBS + 1% BSA, was added to all wells and incubated for 1 h at room temperature. All wells were then washed and dried and observed with an EVOS fluorescent microscope (ThermoFisher Scientific, Waltham, MA, USA). Mock-infected and SARS-CoV-2-infected Vero-E6/TMPRSS2 cells were used as negative and positive controls, respectively.

### Virus neutralizing antibodies

A microneutralization assay was used to determine SARS-CoV-2 virus neutralizing antibody titers from cattle sera as previously described [36]. Briefly, cattle serum was diluted 1:4 and heat-inactivated at 56°C for 30 min while shaking. In duplicate wells, 100 µL of serum was combined with 100 µL of culture media and subjected to 2-fold serial dilutions starting at 1:8 through 1:1024. Separately, SARS-CoV-2 Delta and Omicron BA.2 virus stocks were diluted to 1000 TCID_50_/mL and 100 µl added to 100 µl of the sera dilutions and incubated for 1 h at 37°C. Following incubation, 200 µl of the virus/serum mixture was transferred to 96-well cell culture plates seeded at approximately 2 × 10^5^ cells/mL with low passage Vero-E6/TMPRSS2 cells. Serum with known SARS-CoV-2 neutralizing antibody titer was used as a positive control. Virus only and media only wells were also included. The highest serum dilution at which at least 50% of wells showed virus neutralization (NT_50_) based on the appearance of CPE observed under a light microscope at 96-hours was recorded as the final neutralizing antibody titer.

In a similar manner, serum samples were assayed for neutralizing antibodies against the bovine coronavirus (BCV) Mebus strain (GenBank: U00735.2). Since CPE is not clearly visible with this virus, IFA was used to visualize the presence or absence of neutralizing activity. The IFA procedure was performed as described above for SARS-CoV-2, with the substitution of a BCV specific primary antibody, Z3A5, a monoclonal antibody that targets the spike protein subunit of BCV, kindly provided by Roman Pogranichniy of KSU.

Further analysis of cattle serum was carried out using a commercial SARS-CoV-2 surrogate virus neutralization test kit following the manufacturer’s instructions (GenScript, Piscataway, NJ).

### Detection of antibodies by ELISA

Indirect ELISAs using in-house produced recombinant SARS-CoV-2/Delta receptor-binding domain (RBD) of the spike protein and SARS-CoV-2/Wuhan-like nucleocapsid (N) proteins were used to detect SARS-CoV-2 specific antibodies in cattle sera, as described previously [37]. Briefly, wells were coated with 100 ng of the RBD or N proteins in 100 µL per well coating buffer and incubated overnight at 4°C. Plates were washed twice with PBS-T (0.5% Tween-20 in PBS), blocked with 200 µL per well casein blocking buffer (Sigma-Aldrich) and incubated for 1 h at room temperature followed by a wash with PBS-T. Serum was diluted 1:400 in casein blocking buffer and 100 µL was added to the ELISA plate and incubated for 1 h at room temperature. Wells were then washed three times with PBS-T. An HRP-labelled goat anti-bovine IgG (H + L) secondary antibody was diluted 1:10,000 and 100 µL was added to each well and incubated 1 h at room temperature. Following five washes with PBS-T, 100 µL of TMB ELISA Substrate Solution (Abcam, Cambridge, MA, USA) was added to all wells. After incubation at room temperature for 5 min, the reaction was stopped with 100 µL of stop solution. The optical density (OD) of the ELISA plates was read at 450 nm on an ELx808 BioTek plate reader (BioTek, Winooski, VT, USA). Bovine serum collected in 2014 was used as negative control to determine the positive cut-off value for these assays. The cut-off value was defined by the average OD of the negative serum + 3X standard deviation. Everything above this cut-off was considered positive.

Further analysis of cattle serum was carried out using the commercial ID-Vet ID Screen SARS-CoV-2 Double Antigen Multi-Species Test Kit (Innovative Diagnostics), targeting the N protein, per manufacturer’s instructions.

### Histopathology (H&E)

After 7 days in 10% neutral-buffered formalin, tissues were transferred to 70% ethanol (ThermoFisher) prior to trimming for paraffin embedding. Bony tissues from the respiratory tract (i.e. nasal cavity, rostral, middle and ethmo turbinates) were decalcified with Immunocal™ Decalcifier (StatLab, McKinney, TX), diluted 1:2 in nanopure water, for 5 days at room temperature with agitation prior to trimming and paraffin embedding. Tissues were routinely processed and stained with hematoxylin and eosin (H&E) following standard procedures at the Kansas State Veterinary Diagnostic Laboratory (KSVDL). Veterinary pathologists (unbiased to the treatment groups) examined the slides and provided pathological descriptions. Tissues from naïve calves were used as negative controls for histopathological analysis including immunohistochemistry (IHC) and RNAscope® *in situ* hybridization (ISH), performed at the Louisiana Animal Disease Diagnostic Laboratory (LADDL).

### SARS-CoV-2 specific immunohistochemistry (IHC)

IHC was performed as previously described [33] on four-micron sections of formalin-fixed paraffin-embedded (FFPE) tissue mounted on positively charged Superfrost® Plus slides and subjected to IHC using a SARS-CoV-2-specific anti-nucleocapsid rabbit polyclonal antibody. Lung sections from a SARS-CoV-2-infected hamster were used as positive assay controls.

### SARS-CoV-2-specific RNAscope® in situ hybridization (RNAscope® ISH)

For RNAscope® ISH, an anti-sense probe targeting the spike protein gene (S; nucleotide sequence: 21,563–25,384) of SARS-CoV-2, USA-WA1/2020 isolate (GenBank accession number MN985325.1) was used as previously described (Advanced Cell Diagnostics [ACD], Newark, CA, USA). Five-micron sections of FFPE tissue were mounted on positively charged Superfrost® Plus Slides (VWR, Radnor, PA). The RNAscope® ISH assay was performed using the RNAscope 2.5 LS Duplex Reagent Kit (Advanced Cell Diagnostics, Newark, CA) on the automated BOND RXm platform (Leica Biosystems, Buffalo Grove, IL) with modifications for single-plex detection. Tissue sections were subjected to automated baking and deparaffinization followed by heat-induced epitope retrieval (HIER) using a ready-to-use EDTA-based solution (pH 9.0; Leica Biosystems) at 95 °C for 15 min. Subsequently, tissue sections were treated with a ready-to-use protease (RNAscope® 2.5 LS Protease) for 15 min at 40 °C followed by a ready-to-use hydrogen peroxide solution for 10 min at room temperature. Slides were then incubated with the SARS-CoV-2 S-specific probe mixture for 2 h at 40 °C. The signal was amplified using amplifiers 1 through 3 (AMP1 through AMP3) followed by AMP8 through AMP10 as recommended by the manufacturer. The signal was subsequently detected by incubating with 3,3'-diaminobenzidine (DAB) for 20 min and the BOND DAB Enhancer (Leica Biosystems) for an additional 20 min at room temperature. Slides were counterstained with a ready-to-use hematoxylin for 5 min, followed by a ready to use bluing solution for 2 min. Slides were finally rinsed in deionized water, dried in a 60 °C oven for 30 min, and mounted with Ecomount® (Biocare, Concord, CA, USA). Lung tissue from a SARS-CoV-2-infected Syrian hamster was used as a positive assay control.

## Results

### Clinical evaluations and detection of common bovine pathogens

Prior to the challenge with SARS-CoV-2, calves were examined by a veterinarian. One calf, HT9, was suspected to have mild respiratory signs, and elevated body temperature, cough, and nasal discharge were noted. Coughing was also noted in calf 678 during the acclimation period. Therefore, nasal swabs collected at −1 DPC, were submitted to the KSVDL for the Bovine Respiratory Bacterial and Viral Panel PCR testing (Supplementary Table 1). The results revealed that all calves were negative for bovine coronavirus but were positive for influenza D virus (IDV), *Mannheimia haemolytica*, and *Pasteurella multocida*. Based on this information, RNA extracted from respiratory and lymphoid tissues at 3 DPC was pooled and tested via RT-qPCR for the presence of IDV. RNA from both SARS-CoV-2 and IDV were detected in the nasal turbinate and trachea/bronchi of calf HT1 and the mandibular lymph node of calves HT1 and 678 (Supplementary Table 2); the IDV positive animal 678 was showing respiratory signs before SARS-CoV-2 challenge.

Following SARS-CoV-2 challenge, health checks and rectal temperatures were recorded daily. Coughing was documented in calf HT6 at 4 and 14 DPC, and coughing and nasal discharge were noted in calf HT9 at 5 and 18 DPC. Rectal temperatures did not exceed 103.4°F during the study period in any of the animals enrolled in the study (data not shown).

### Detection of SARS-CoV-2 RNA in clinical samples and tissues

**Figure 2.**
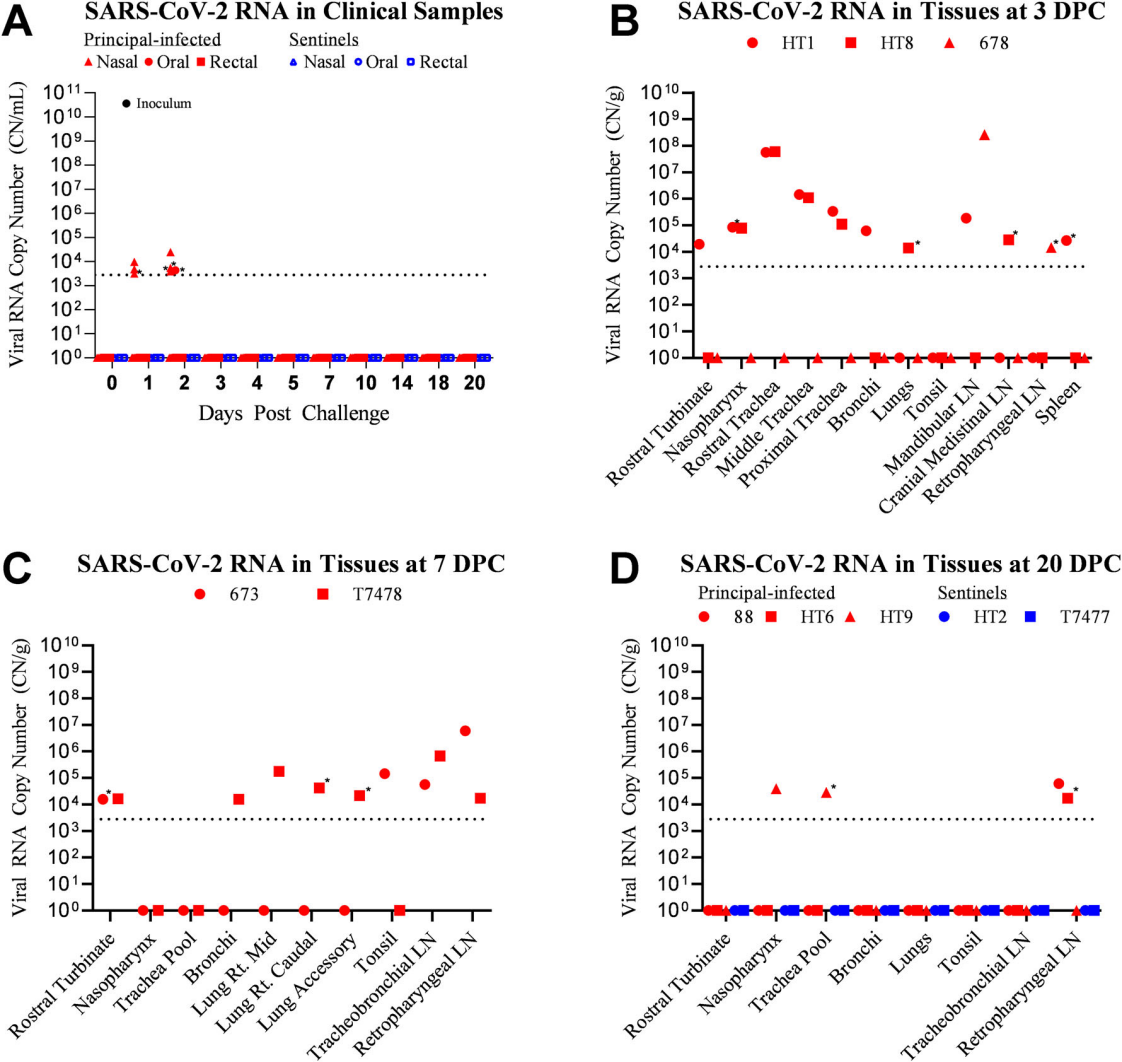
Detection of SARS-CoV-2 RNA in clinical samples and tissues. RT-qPCR was performed on nasal, oral, and rectal swabs collected from principal-infected and sentinel calves (A), and on various tissues homogenates of calves euthanized at 3 (B), 7 (C), and 20 (D) days post-challenge (DPC) to detect the presence of SARS-CoV-2 specific RNA. Mean (n = 2) viral RNA copy number (CN) per mL (A) or per gram of tissue (B, C, D) is reported based on the detection of SARS-CoV-2 nucleocapsid gene in individual animals. Only a subset of tissues which were collected and tested are represented in this figure. Asterisks (*) indicate samples with one of two RT-qPCR reactions positive. The limit of detection for this RT-qPCR assay is indicated by the dotted lines.

Clinical samples, including nasal, oral, and rectal swabs, and whole blood were tested for the presence of SARS-CoV-2 RNA by RT-qPCR assay (Figure 2A). Samples which were collected prior to challenge (−1 DPC) were negative for SARS-CoV-2 RNA. Six of the eight challenged calves shed SARS-CoV-2 RNA during the first 2 DPC (Figure 2A). At 1 DPC, three out of eight challenged calves (673, 678, 88) had SARS-CoV-2 RNA present in nasal swabs; all oral swabs and rectal swabs were negative. At 2 DPC, three calves (HT1, HT9, HT6) from the primary challenge group, had SARS-CoV-2 RNA present in nasal swabs. One calf (HT1) shed SARS-CoV-2 RNA from the oral cavity at 2 DPC. Whole blood and all swabs collected after 2 DPC (3, 4, 5, 7, 10, 14, 18, 20 DPC) were negative for the presence of SARS-CoV-2 RNA. No SARS-CoV-2 RNA was detected in swabs or blood collected from sentinel calves (n = 2; T7477 and HT2) at any time point.

Three calves from the principal-infected group (HT1, HT8, 678) were euthanized at 3 DPC, and another two calves (673, T7478) from this group were euthanized at 7 DPC to evaluate SARS-CoV-2 distribution in tissues and pathological alterations during different stages of infection. At 3 DPC, SARS-CoV-2 RNA was present in upper respiratory tissues of two calves (HT1 and HT8) (Figure 2B). The spleen, mandibular, cranial mediastinal, and retropharyngeal lymph nodes were also positive for SARS-CoV-2 RNA in some animals at 3 DPC. At 7 DPC, SARS-CoV-2 RNA was present in the nasal turbinates, as well as tracheo-bronchial, and retropharyngeal lymph nodes of calves 673 and T7478 (Figure 2C). The tonsil of calf 673 was also positive at 7 DPC. Additionally, three lung lobes (right middle, right caudal, and accessory) and the bronchi of calf T7478 had SARS-CoV-2 RNA present at 7 DPC.

The five remaining calves (three principal-infected animals and two sentinels) were euthanized at 20 DPC. Of the principal-infected group, two calves (88, HT6) had SARS-CoV-2 RNA present in the retropharyngeal lymph node (Figure 2D). The nasopharynx and trachea of principal-infected calf HT9 were also positive for SARS-CoV-2 RNA at 20 DPC. There was no SARS-CoV-2 RNA detected in any tissues of the two sentinel calves (HT2, T7477) evaluated at 20 DPC.

### Competition between SARS-CoV-2 Delta and Omicron BA.2 VOCs in co-infected calves

**Figure 3.**
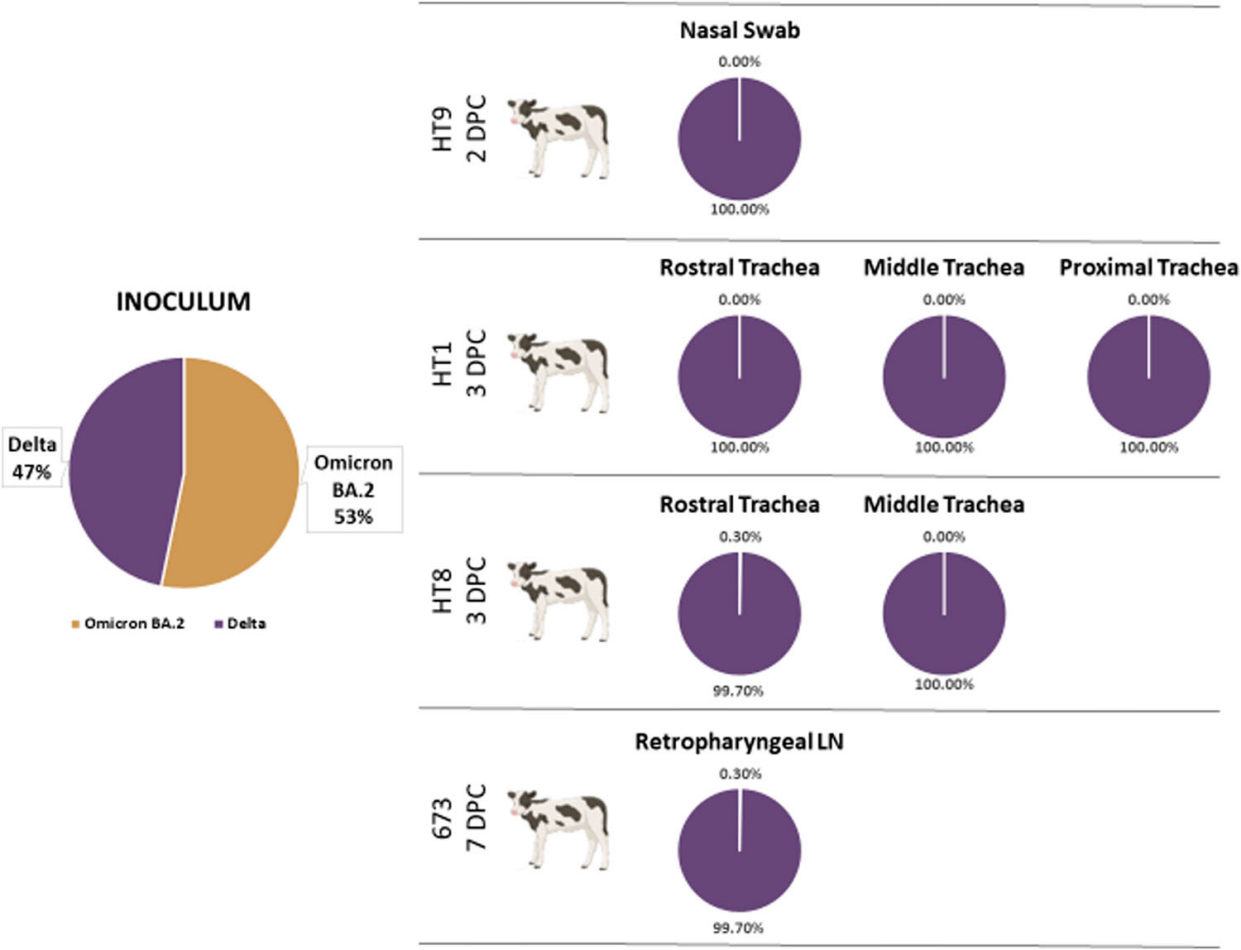
Identification of SARS-CoV-2 variants of concern in nasal swab and tissues. Next-generation sequencing was used to determine the percent composition of the SARS-CoV-2 variants of concern Delta and Omicron BA.2, present in the inoculum, swab samples and tissues collected from the co-infected calves. Reads were analyzed using the VirStrain (Version 1.12) software package to determine the genetic composition (% of each lineage) of SARS-CoV-2 RNA present in samples. The genome sequences from the virus stocks used in our challenge experiment were used to construct the reference database for VirStrain lineage assignment.

Calves were challenged with a 50/50 mixture of SARS-CoV-2 Delta and Omicron BA.2 VOCs. To evaluate the *in vivo* competition between these two strains, next-generation sequencing (NGS) was used in conjunction with the VirStrain software package to identify the proportion of each virus strain present in the inoculum as well as clinical samples, and tissues which contained SARS-CoV-2 RNA (Figure 3). The inoculum contained 53.2% Omicron BA.2 VOC and 46.8% Delta VOC. Of the seven nasal/oral swabs which were positive on 1 and 2 DPC, only one nasal swab at 2 DPC from calf HT9 had sufficient sequencing coverage and depth to provide reliable results; the nasal swab of calf HT9 at 2 DPC was 100% Delta VOC (Supplementary Table 3). Trachea samples collected at 3 DPC from calves HT1 and HT8 were also analyzed by NGS and found to be 100% Delta VOC (Figure 3). Additionally, one retropharyngeal lymph node collected at 7 DPC from calf 673 was also found to be 100% Delta VOC.

### Isolation of virus from tissues

The presence of SARS-CoV-2 RNA in clinical samples and tissues collected from the calves warranted further evaluation for the presence of infectious virus. Samples with Ct values < 30 were selected as candidate samples for virus isolation. This included tissue homogenates produced from the rostral trachea sections of calves HT1 and HT8 at 3 DPC, the mandibular lymph node from calf 678 at 3 DPC, and the retropharyngeal lymph node from calf 673 at 7 DPC. Homogenates were divided among 12 replicate wells for each sample. Of the twelve replicates, two wells of the rostral trachea from calf HT1 showed CPE, and three wells of the mandibular lymph node from calf 678 showed CPE after a single passage on Vero-E6/TMPRSS2 cells. All wells were fixed and subjected to IFA for confirmation of SARS-CoV-2 antigen presence (Supplementary Figure 1). The wells with CPE were IFA positive; none of the wells without CPE, or negative control wells, were fluorescence positive, suggesting there was no SARS-CoV-2 replication in these wells. NGS analysis of the Delta VOC virus isolated from the rostral trachea of calf HT1 showed no changes in the RBD region of the S gene sequence compared to the sequence of the SARS-CoV-2 Delta inoculum. The sequence obtained from the mandibular lymph node of calf 678 had insufficient coverage to make a detailed assessment.

### ELISAs

**Figure 4.**
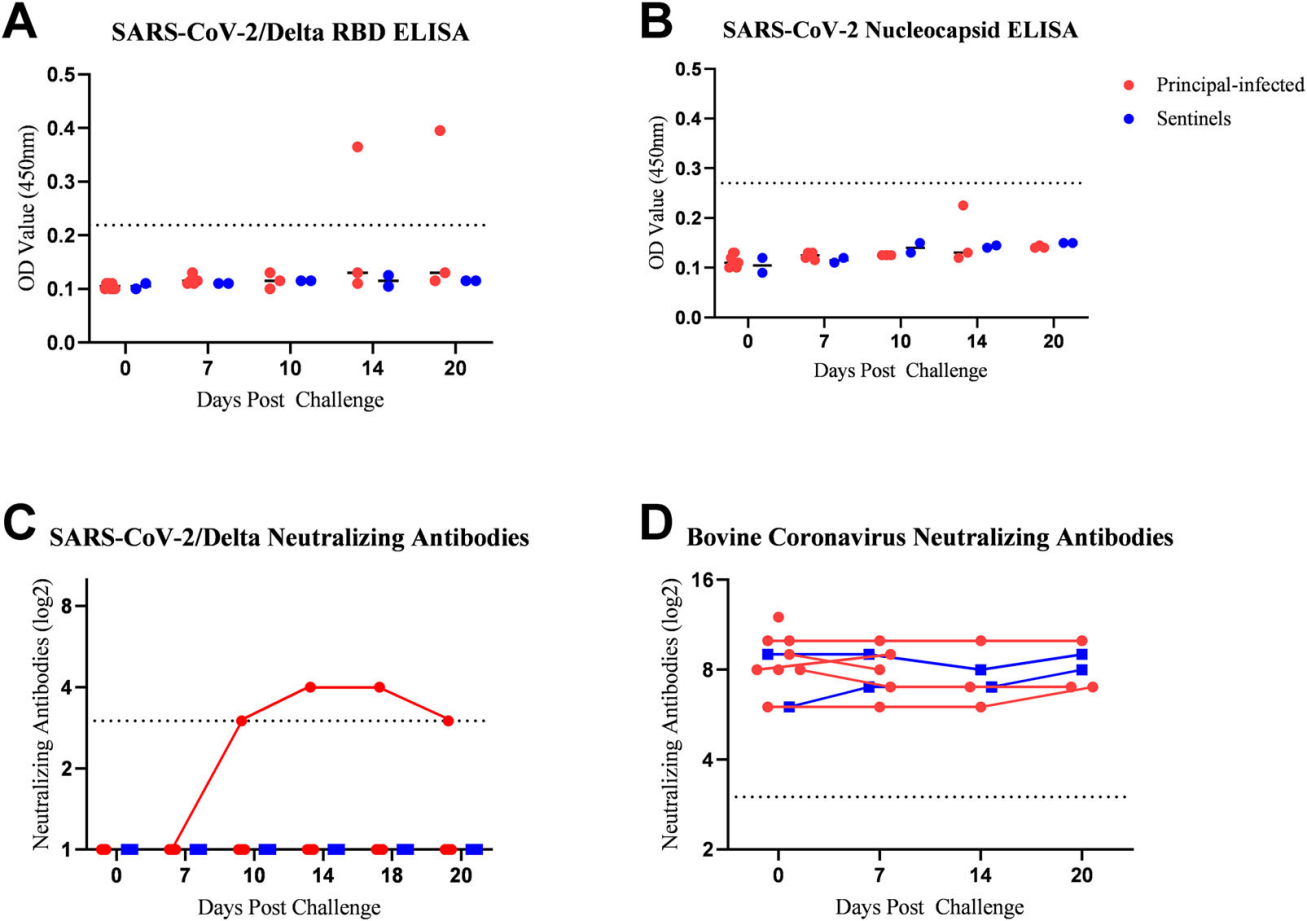
Serology of SARS-CoV-2 infected calves. Indirect ELISAs using in-house produced recombinant SARS-CoV-2/Delta receptor-binding domain (RBD) of the spike protein (A) and SARS-CoV-2/Wuhan-like nucleocapsid (N) protein (B), for detection of SARS-CoV-2 specific antibodies in cattle sera (Bold et al. 2022). The cut-off (dotted line) was determined by the average OD value of negative serum samples (n = 12) collected from cattle in 2014 + 3X the standard deviation. Serum was also tested for the presence of SARS-CoV-2 neutralizing antibodies using a classic virus neutralization assay employing the Delta VOC (C) or Omicron BA.2 VOC (data not shown) with serum dilutions starting at 1:8 (dotted line). Data is represented as log2 of the reciprocal of the dilution where one of two replicates exhibited 100% neutralization of the input virus (1000 TCID_50_/mL). Likewise, serum was tested for the presence of neutralizing antibodies for bovine coronavirus (D) using the Mebus strain of Bovine Coronavirus.

Indirect ELISAs, developed in-house, targeting the SARS-CoV-2/Delta RBD and SARS-CoV-2/Wuhan-like N were used to screen cattle serum for SARS-CoV-2-specific antibodies. All serum was negative for antibodies against the SARS-CoV-2 RBD and N protein prior to challenge. SARS-CoV-2/Delta RBD-specific antibodies SARS-CoV-2 were detected at 14 and 20 DPC in calf HT9 (Figure 4A). Surprisingly, antibodies against the SARS-CoV-2 N protein were not detected by ELISA in any cattle during the study period of 20 days, although calf HT9 had elevated OD readings at 14 DPC; however it was below the positive cut-off value (Figure 4B). The above results were confirmed with the ID-Vet ID Screen SARS-CoV-2 Double Antigen Multi-Species Test Kit targeting the SARS-CoV-2 N protein (data not shown). The sentinel calves did not produce ELISA-positive RBD- or N-specific antibodies during the entire study period.

### Virus neutralizing antibodies

Serum was tested for the presence of neutralizing antibodies against both, SARS-CoV-2 Delta and Omicron BA.2, as well as bovine coronavirus. No SARS-CoV-2 neutralizing antibodies were present prior to challenge. At 10 DPC, one of three remaining principal-infected calves, animal HT9, had developed neutralizing antibodies against SARS-CoV-2 Delta, but not against the Omicron BA.2 virus; the neutralizing antibody titer was maintained until the termination of the study at 20 DPC (Figure 4C). The sentinel calves did not produce neutralizing antibodies against either SARS-CoV-2 VOC during the entire study period. These results were confirmed using a commercial SARS-CoV-2 surrogate virus neutralization test kit (GenScript; data not shown). All calves (principal-infected and sentinels) had neutralizing antibody titers against bovine coronavirus prior to challenge, which persisted through the study period; no significant increase was observed during the study period (Figure 4D).

### Gross pathology

On postmortem examination at 3 DPC, the only significant gross lesions observed were present in the lung of principal-infected calf HT1 and consisted of mild multifocal consolidation of the right cranial and right and left middle lung lobes, affecting less than 5% of the total lung parenchyma. No significant gross lesions were observed for either of the calves euthanized at 7 DPC. At 20 DPC, one principal challenged calf, animal HT9, had mild to moderate multifocal consolidation of the right cranial lobe affecting less than 5% of the total lung parenchyma; no significant gross lesions were observed for the other two principal-infected or the two sentinel calves.

### Histopathology

**Figure 5.**
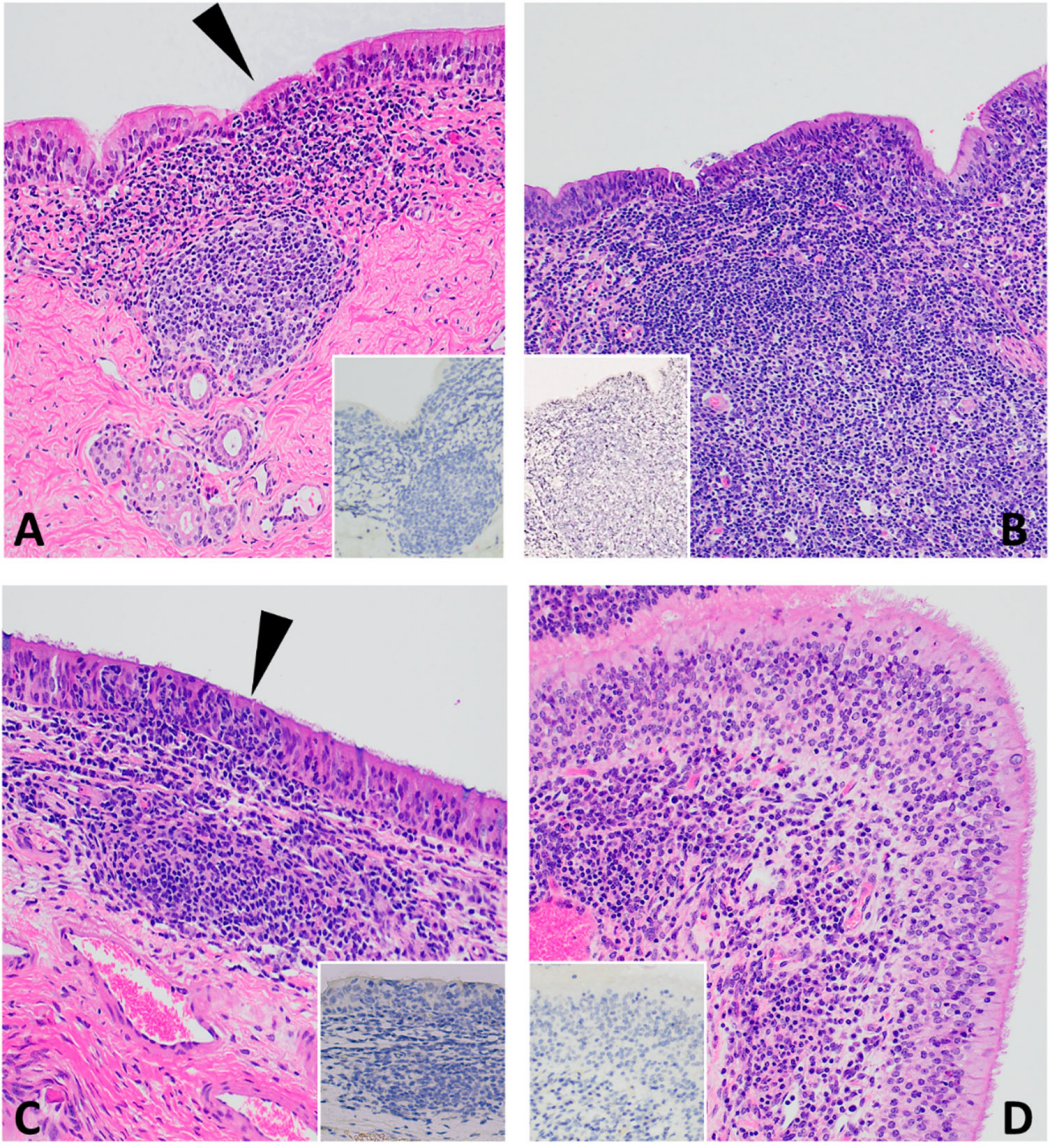
Histopathology of 3 DPC respiratory tissues from calf HT1. At 3 DPC, (A) in the trachea, there was mild and segmental attenuation of the respiratory epithelium with loss of cilia, lymphocyte and/or neutrophil transmigration through the epithelium, individual cellular degeneration and necrosis, and occasional accumulation of cellular debris on the epithelial surface (arrowhead). Dense sheets of mononuclear inflammatory cells expanded into the lamina propria and commonly follicular aggregates of lymphocytes, plasma cells and macrophages arranged into loose aggregates or partially organized follicular structures; SARS-CoV-2 antigen was not detected by IHC (insert). (B) Similar segmental attenuation of the respiratory epithelium was noted in the bronchi with cellular degeneration/necrosis of individual epithelial cells, lymphocytic and neutrophilic transmigration. Loose infiltrates of lymphocytes and a lesser number of neutrophils were observed in the superficial mucosa and mixed lymphocytic and histiocytic infiltrates, many arranged in follicular aggregates expanding into the superficial and deep lamina propria; SARS-CoV-2 viral antigen was not detected (insert). (C) The rostral turbinates were characterized by lymphoplasmacytic or neutrophilic rhinitis with epithelial transmigration of inflammatory cells (arrowhead) and segmental loss of cilia. Mixed lymphocytic and histiocytic inflammation multifocally expanded the subjacent lamina propria and the interstitium separating submucosal glands. IHC for SARS-CoV-2 viral antigen was negative (insert). (D) The olfactory mucosa contained similar lymphoplasmacytic or histiocytic inflammation within the lamina propria and the interstitum separating submucosal glands; SARS-CoV-2 viral antigen was not detected (insert). H&E and Fast Red, 40× total magnification.

**Figure 6.**
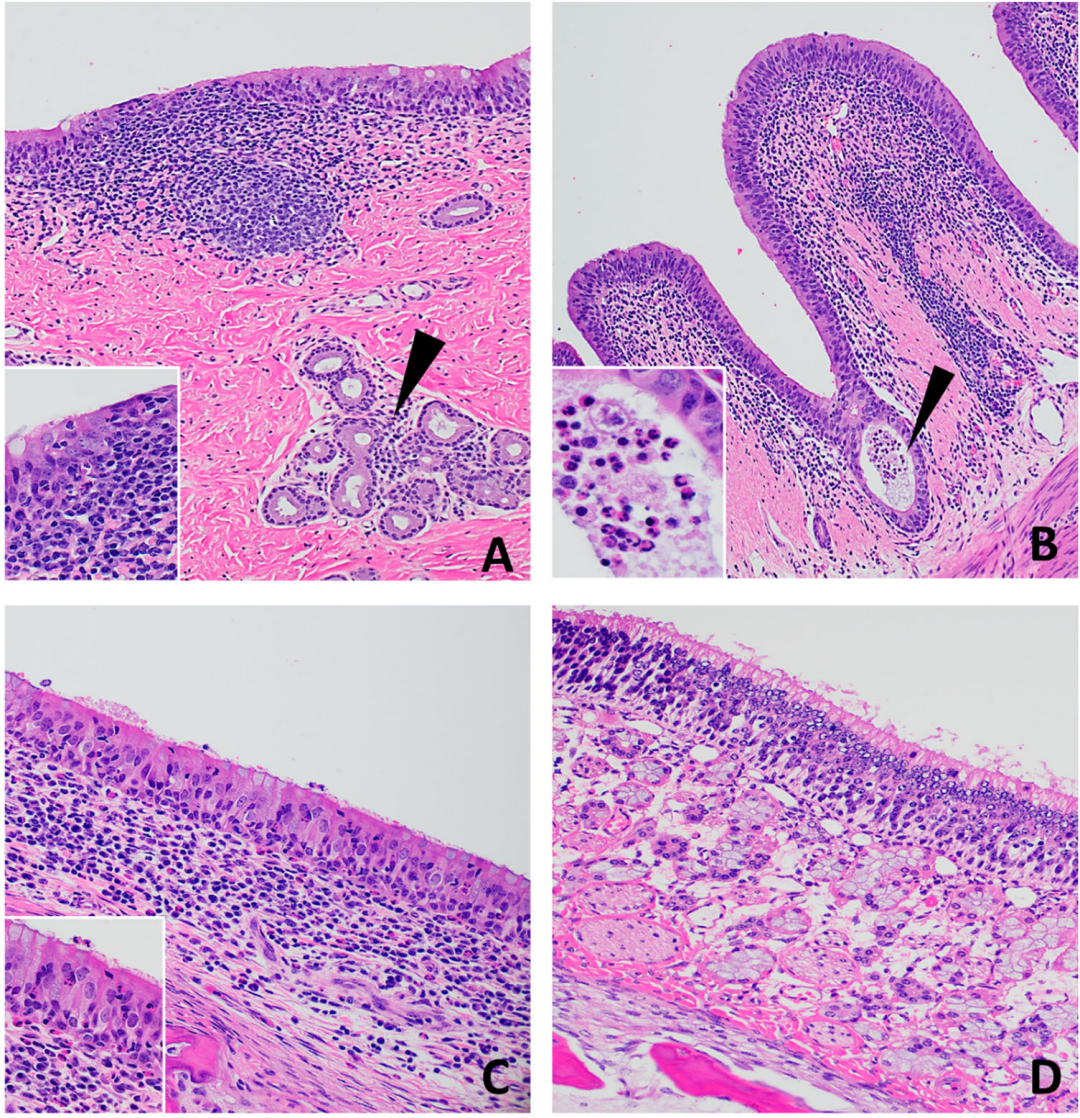
Histopathology of 7 DPC respiratory tissues from calf 673. (A) There was moderate, segmental attenuation and disorganization of the respiratory epithelium with loss of cilia, moderate lymphocyte and/or neutrophil transmigration and individual cellular degeneration and necrosis (Insert). Mononuclear inflammatory cells expanded the lamina propria and were arranged in dense follicular aggregates. Inflammatory cells (lymphocytes and plasma cells) also infiltrated the interstitium between submucosal glands (arrowhead). (B) Mild segmental attenuation of the respiratory epithelium in the bronchi with infrequent cellular degeneration/necrosis. Cellular debris and degenerate neutrophils sporadically accumulated in the lumen or on the epithelial surface (arrowhead and insert). Lymphocytes, plasma cells, macrophages, and lesser numbers of neutrophils expanded the submucosa and extended between submucosal glands. (C) The rostral turbinates had lymphoplasmacytic and neutrophilic infiltrates with epithelial transmigration of inflammatory cells onto the epithelial surface with segmental loss of cilia (Insert). Loose and dense sheets of mixed lymphocytes, histiocytes, and neutrophils infiltrated the subjacent lamina propria and between glands. (D) The olfactory mucosa of the ethmoturbinates was normal. H&E, 40-400× total magnification.

Histological evaluation was performed on tissue sections from all organs collected during postmortem examination at 3, 7 and 20 DPC for all animals in the study. IHC for viral antigen and RNAscope® ISH for viral RNA was performed on selected tissues (nasal turbinates, nasopharynx, trachea, lung and selected lymph nodes) from all animals at 3, 7 and 20 DPC. Histological lesions were characterized by mild to moderate, multifocal, non-suppurative inflammation with mild to severe lymphoid hyperplasia in the upper respiratory tract including the nasal turbinates, oropharynx, trachea, and bronchi. Representative histological lesions at 3, 7, and 20 DPC are provided in Figure 5, Figure 6, and Supplementary Figure 2, respectively. Histological changes in calves HT1 and 678 at 3 DPC and calves T47478 and 673 at 7 DPC were the most prominent and represent active inflammatory lesions. Overall, lymphoid hyperplasia was present in the form of dense aggregates of lymphocytes, plasma cells, and macrophages commonly arranged as immature follicular structures in the submucosa at 3 DPC throughout the respiratory tract; they became more organized and mature and occurred among, and extended deeper up to, the submucosal glands by 7 DPC. Turbinate sections and nearly all tracheal sections had mild to moderate segmental inflammation in the respiratory epithelium and submucosa that was most prominent at 3 and 7 DPC (Figure 5 and Figure 6). Inflammation was observed predominantly within the superficial submucosa, as loose aggregates or dense sheets of mononuclear cells. Submucosal edema was infrequent, occurring segmentally in regions where inflammatory cells infiltrated the epithelium. Cellular infiltrates in the respiratory epithelium were observed as individual or small clusters of primarily mononuclear cells. Neutrophilic infiltrates were infrequent and observed scattered in the respiratory epithelium and subjacent submucosa commonly in foci where epithelial changes and submucosa edema were noted. The epithelial changes were characterized by mild to moderate attenuation of the epithelium with disorganization, thinning and flattening of the epithelium with occasional segmental loss of cilia (Figure 5, Figure 6A and inserts). Epithelial necrosis, when present, was observed as shrunken or vacuolated cells with karyorrectic and pyknotic nuclei. Occasionally, inflammation and cellular degradation was observed in the glandular epithelium. Similar inflammation, albeit less severe, was observed in sections of the nasal turbinates and ethmoturbinates (Figure 5 and Figure 6; panels C and D). Cellular debris and inflammatory cells were infrequently found in small clusters and as individual cells within the airway lumen and commonly attached to the cilia (Figure 6B and insert, and 6C).

Inflammation in primary and secondary bronchi was most pronounced at 3 DPC and was characterized by loose infiltrates of lymphocytes, macrophages and plasma cells in the submucosa that was markedly expanded by multifocal to coalescing lymphoid follicles (follicular hyperplasia), similar to the changes seen in the trachea. Occasional clusters of individual neutrophils were observed scattered within the epithelium and submucosa. Loose, less severe inflammation was observed in the submucosa, accompanied by mild follicular hyperplasia at 7 DPC (Figure 6) and 20 DPC (Supplementary Figure 2).

By 20 DPC, the microscopic alterations were most consistent as lymphoid hyperplasia with minimal epithelial changes (Supplementary Figure 2). IHC and ISH performed on selected tissue sections with lesions (turbinates, trachea, nasopharynx, lung and lymph nodes) from cattle at 3, 7 and 20 DPC failed to identify viral antigen or viral RNA in the lesions despite these tissues being RT-qPCR positive for viral RNA and some being positive by virus isolation.

### Lung histology

At 3 DPC, histological evaluation of calf HT1 lung sections with gross evidence of consolidation revealed compressed and congested alveoli (atelectasis). Lung sections from calf HT8 demonstrated several areas of atelectasis in the right cranial lung lobe. As mentioned above, IHC for SARS-CoV-2 antigen was negative. Lung sections from calf 678 from the right middle and caudal lung lobes had multiple regions of chronic-active, purulent broncho-interstitial pneumonia with abscess formation and intralesional coccobacillary bacteria. These regions were characterized by central foci of necrosis, mineralization, and large numbers of degenerate inflammatory cells bordered by highly vascularized granulation tissue which in turn was bordered by dense layers of fibrosis (abscess); adjacent lobules were compressed and had alveoli filled with fibrin and mixed inflammatory cell infiltrates; in some regions small coccobacilli occurred in alveolar spaces. IHC for SARS-CoV-2 antigen was negative as was RT-qPCR. Lung lesions were most consistent with a chronic-active bacterial infection.

Aside from the inflammatory changes described in bronchi (see above and Figure 6 and Supplementary Figure 2), no significant lesions were seen in the lungs of cattle at 7 and 20 DPC.

## Discussion

Cattle are an important food source globally and critical for economic stability for a large fraction of the world’s population. Humans have a long history of dependency on cattle and this close relationship creates opportunities for zoonotic spillover. Human coronavirus OC43, a betacoronavirus which accounts for 10-30% of common colds in humans, is believed to have spilled over from cattle to humans relatively recently [38,39]. Interestingly, a high seroprevalence of influenza D virus (IDV) amongst cattle workers has been reported which is suggestive of extensive exposure of these workers to IDV from cattle [40]. Evidence of previous cross-species viral transmission between cattle and humans and the close interaction between them provides the rationale for continued evaluation of cattle for their susceptibility to SARS-CoV-2 and other zoonotic pathogens.

In the present study, we evaluated the susceptibility and transmission of two SARS-CoV-2 variants of concern, Delta and Omicron BA.2, in 4-month-old male Holstein calves. During the 20-day study period, clinical samples were collected, and tissues were harvested from principal-infected and sentinel calves which were euthanized at 3 (*n* = 3 principal-infected), 7 (*n* = 2 principal-infected), and 20 DPC (*n* = 5; three principal-infected, two sentinels). The results of this study indicate that calves have low susceptibility to the SARS-CoV-2 Delta VOC, which outcompeted the Omicron BA.2 VOC; these data also suggest that calves might not be permissive to Omicron VOC BA.2 infection. Evidence for this is provided by next-generation sequencing (NGS) data, which revealed the presence of SARS-CoV-2 Delta VOC but not the Omicron BA.2 VOC in nasal swabs and tissues. Additionally, the neutralizing antibodies present in the blood of principal-infected calves were generated specific for the SARS-CoV-2 Delta VOC, but not the Omicron BA.2 VOC. Virus isolated from tracheal and lymphoid tissues from two calves at 3 DPC confirmed the presence of an active infection with the Delta VOC.

Previous SARS-CoV-2 challenge studies in cattle, which used Wuhan-like SARS-CoV-2 isolates including the TGR1/NY/20 tiger isolate, with a similar administration route and dose range as in our study, resulted in very limited infection and only transient serological responses [23–25]. Several findings from our study with the Delta and Omicron BA.2 VOCs are different from previous experiments. First, SARS-CoV-2 RNA was detected in nasal and/or oral swabs in our study from the majority of principal-infected animals (six out of eight challenged calves, i.e. 75%) within the first 2 DPC, compared to only 33% of calves in the other studies [23–25]. Second, we observed that out of the 31 fresh tissue samples which were collected during postmortem examination at 3 DPC from principal-infected calves, 12 tissues (36%) were positive for SARS-CoV-2 RNA. In addition, infectious virus was isolated from the rostral trachea section of one calf and the mandibular lymph node of another one at 3 DPC. Furthermore, at 7 DPC, SARS-CoV-2 RNA was still detectable in 8/26 tissues (31%) collected from two principal-infected calves. SARS-CoV-2 RNA was also detected in 3/26 tissues (12%) which were collected at 20 DPC from the remaining principal-infected calves. In contrast, Falkenberg et al. [24] reported detection of SARS-CoV-2 RNA only in the tracheo-bronchial lymph node of one calf at 9 DPC and did not isolate any virus from swabs or tissues during the entire study; however, SARS-CoV-2 RNA was detected using in situ hybridization. Bosco-Lauth et al. also isolated SARS-CoV-2 from the trachea of 1/3 challenged cattle at 3 DPC, although 9 other tissues, including those of the respiratory tract, systemic organs and lymph nodes, were negative for infectious virus as well as SARS-CoV-2 RNA. Previous studies reported only transient serological responses in SARS-CoV-2 challenged cattle. In contrast, one out of three principal-infected calves which were maintained beyond 7 DPC in our study, seroconverted and maintained neutralizing antibody titers until the study termination on 20 DPC. Overall, the present study resulted in a greater proportion of calves shedding SARS-CoV-2 RNA and harboring viral RNA in tissues, with one of the three principal-infected calves remaining beyond 7 DPC developing and maintaining a neutralizing antibody response. These findings may indicate that cattle are more susceptible to SARS-CoV-2 Delta VOC than the Wuhan-like SARS-CoV-2 isolates which have been used for cattle infection in previous studies [23–25].

Similar to the study by Ulrich et al. [25], all calves enrolled in our study had varied levels of neutralizing antibody titers against bovine coronavirus (BCV), although we did not detect any BCV-specific RNA in nasal swabs collected prior to challenge; this suggests that there was no active BCV infection in the animals enrolled in the study. Consistent with what has been previously reported by Ulrich and colleagues [25], we did not see any cross-protection provided from prior BCV infection [25]. One calf (HT9) in our study had clinical symptoms consistent with mild Bovine Respiratory Disease (BRD) complex prior to SARS-CoV-2 challenge. Interestingly, this calf was the only animal that seroconverted and had low levels of SARS-CoV-2 RNA present in upper respiratory tissues at 20 DPC. This may suggest that the health and immune status of this animal at the time of SARS-CoV-2 challenge might have influenced the course of disease. Previous studies have determined that health status can affect expression and distribution of host ACE2 receptors [41,42]; this may have contributed to different disease progression in this calf. Additionally, all calves were found to have an underlying infection with influenza D virus (IDV), evident by positive RT-qPCR from nasal swabs collected pre-challenge. Influenza D virus (IDV) is a newly described virus of cattle, not commonly associated with the BRD complex but was included in this diagnostic panel as IDV has recently been detected in approximately 12% of clinical samples submitted for BRD testing at KSVDL. Further testing revealed the presence of IDV RNA and SARS-CoV-2 RNA in the same respiratory and lymphoid tissues collected at 3 DPC (Supplementary Table 2). IDV can be an incidental finding in cattle or occasionally may present as tracheitis [43,44]. Histological evidence of acute inflammation in the turbinates, trachea and bronchi at 3 DPC with continuation and maturation of the inflammation and lymphoid hyperplasia at 7 and 20 DPC in these animals are consistent with previous SARS-CoV-2 lesions in permissive species as well as those that are less permissive [14,33]. Although attempts were made to localize SARS-CoV-2 antigen or viral RNA within tissues using IHC and ISH, respectively, this testing was negative. However, RT-qPCR did confirm the presence of SARS-CoV-2 RNA in the tissues with lesions. The discrepancy in these results may be attributed to variability in sampling of tissue that was collected for formalin fixation and fresh tissue processing, and the fact that a low number of cells were infected and not present in both sample types. SARS-CoV-2 and IDV have similar target tissues and would be histologically indistinguishable without confirmatory antigen/viral RNA detection within the lesion [33,44]. It is not clear whether respective IDV infection increases, reduces yet or is neutral for the susceptibility to SARS-CoV-2.

Currently, there is no evidence that cattle serve as a primary reservoir or amplifying host for SARS-CoV-2. This is supported by the results of our study and others, in which low levels of SARS-CoV-2 shedding are present only for a short period after infection; and under experimental conditions, there is no onward transmission to sentinel calves. However, there is now serological evidence of naturally infected cattle in Germany and Italy, where sampling periods coincided with the Delta VOC wave of transmission in humans [28,29]. In Germany, Wernike et al. [29] reported that 11/1,000 cattle were antibody-positive when 83 farms were sampled, suggesting these were individual spillover events, likely from infected humans. However, in Italy, Fiorito et al. [28] reported 13/24 lactating cattle which were sampled on a single farm had neutralizing antibodies against SARS-CoV-2; the latter data are suggestive of transmission occurring within the herd. Interestingly, SARS-CoV-2 RNA was isolated from nasal and/or rectal swabs collected from cattle during the human Delta VOC wave of transmission in both India and Nigeria, suggesting Delta VOC susceptibility and recent exposure [27,30]. Together, these data support the conclusion that during the Delta VOC wave of transmission in humans, there were instances of spillover of SARS-CoV-2 Delta VOC from humans into cattle. It is conceivable that spillover had also occurred earlier in the pandemic, however cattle were not included in surveillance efforts at that time. Epidemiological evidence of cattle infected with SARS-CoV-2 only became apparent when enhanced surveillance efforts were put into place as the SARS-CoV-2 pandemic continued and when more focus was placed on surveillance in animals. Most importantly, the findings of our study in conjunction with evidence of naturally infected cattle during the Delta VOC surge in humans, support the conclusion that cattle are more susceptible to SARS-CoV-2 Delta VOC than the previously studied Wuhan-like SARS-CoV-2 viruses. Recently, several studies have provided clear evidence that SARS-CoV-2 VOCs have expanded their host range due to key amino acid substitutions in the RBD of the Spike protein which alter the binding affinity with host ACE2 receptors [17,18,20].

While human to human transmission continues to be the main driver for SARS-CoV-2 transmission, it has become apparent throughout the pandemic that *One Health* measures must be implemented for comprehensive monitoring of SARS-CoV-2 spillover transmissions. Infection of SARS-CoV-2 in cattle, other ruminant species, or the diverse range of mammalian species on the susceptible animal species list [9] provide ample opportunities for viral evolution via adaptive mutations and possibly recombination with other SARS-CoV-2 strains or related animal coronaviruses with the potential to affect both animal and public health. Although cattle do not seem to be highly susceptible to currently circulating strains of SARS-CoV-2, there is the possibility that his might change with future emerging virus variants. Therefore, it is critical that agriculturally important species which have close contact with humans, such as cattle and other domestic and even wild ruminants, are included in national and international surveillance efforts and are evaluated for their susceptibility to newly emergent SARS-CoV-2 VOCs [45].

## Supplementary Material

EMI_Supplementary_SARS2_cattle_Final
