# Supplementary material for: Experimental co-infection of calves with SARS-CoV-2 Delta and Omicron variants of concern
Source: Emerg Microbes Infect. 2023 Nov 8;13(1):2281356. doi: 10.1080/22221751.2023.2281356 (PMC10763854; doi:10.1080/22221751.2023.2281356)
Supplement: EMI_Supplementary_SARS2_cattle_Final [file TEMI_A_2281356_SM7169.docx]

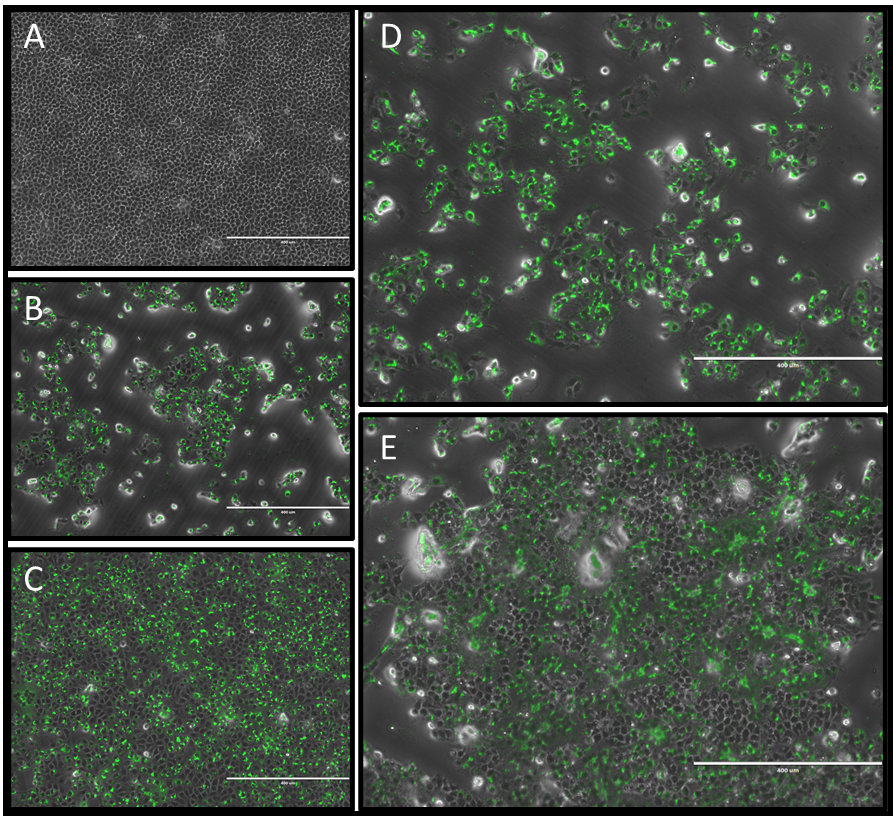


**Supplementary Figure 1. Virus isolation and immunofluorescent staining.** Tissue homogenates with Ct values < 30 were subjected to a single passage on Vero-E6/TMPRSS2 cells. SARS-CoV-2 infection was confirmed with indirect immunofluorescence assay (IFA) staining at 3 days post infection using SARS-CoV-2 N- and RBD-monoclonal antibody supernatants. Wells were then observed at 10x magnification with an EVOS fluorescent microscope. Mock-infected (A), SARS-CoV-2/Delta infected (B), and SARS-CoV-2/Omicron BA.2 infected (C) Vero-E6/TMPRSS2 cells were used as negative and positive controls, respectively. Infectious SARS-CoV-2 was observed in the rostral trachea of calf HT8 (D) and the mandibular lymph node of calf 678 (E) from tissues harvested at 3 DPC.

| **Animal ID** | **Bovine Viral Diarrhea Virus** | **Bovine Herpesvirus 1** | **Bovine Respiratory Syncytial Virus** | **Mycobacterium bovis** |
| --- | --- | --- | --- | --- |
| **673** | NEGATIVE | NEGATIVE | NEGATIVE | NEGATIVE |
| **HT8** | NEGATIVE | NEGATIVE | NEGATIVE | NEGATIVE |
| **HT1** | NEGATIVE | NEGATIVE | NEGATIVE | NEGATIVE |
| **HT9** | NEGATIVE | NEGATIVE | NEGATIVE | NEGATIVE |
| **88** | NEGATIVE | NEGATIVE | NEGATIVE | NEGATIVE |
| **T7478** | NEGATIVE | NEGATIVE | NEGATIVE | NEGATIVE |
| **678** | NEGATIVE | NEGATIVE | NEGATIVE | NEGATIVE |
| **HT6** | NEGATIVE | NEGATIVE | NEGATIVE | NEGATIVE |
| **T7477** | NEGATIVE | NEGATIVE | NEGATIVE | NEGATIVE |
| **HT2** | NEGATIVE | NEGATIVE | NEGATIVE | NEGATIVE |
| **Animal ID** | **Bovine Corona Virus** | **Influenza D Virus** | **Mannheimia haemolytica** | **Pasteurella multocida** |
| **673** | NEGATIVE | 34.99 | 34.90 | 30.71 |
| **HT8** | NEGATIVE | 36.21 | 36.14 | 25.87 |
| **HT1** | NEGATIVE | 32.74 | 37.38 | 30.17 |
| **HT9** | NEGATIVE | 34.00 | 28.24 | 28.02 |
| **88** | NEGATIVE | 36.23 | 36.64 | 28.61 |
| **T7478** | NEGATIVE | 33.67 | 32.54 | 27.17 |
| **678** | NEGATIVE | 21.28 | 32.00 | 27.02 |
| **HT6** | NEGATIVE | 37.24 | 35.57 | 32.55 |
| **T7477** | NEGATIVE | 35.82 | 32.32 | 28.99 |
| **HT2** | NEGATIVE | 36.02 | 37.26 | 31.36 |
| **Animal ID** | **Histophilus somni** | **Bibersteinia trehalosi** |  |  |
| **673** | NEGATIVE | NEGATIVE |  |  |
| **HT8** | NEGATIVE | NEGATIVE |  |  |
| **HT1** | NEGATIVE | NEGATIVE |  |  |
| **HT9** | NEGATIVE | NEGATIVE |  |  |
| **88** | NEGATIVE | NEGATIVE |  |  |
| **T7478** | NEGATIVE | NEGATIVE |  |  |
| **678** | NEGATIVE | NEGATIVE |  |  |
| **HT6** | NEGATIVE | NEGATIVE |  |  |
| **T7477** | NEGATIVE | NEGATIVE |  |  |
| **HT2** | NEGATIVE | NEGATIVE |  |  |

**Supplementary Table 1. Results of bovine respiratory panel PCR.** Swabs collected prior to challenge (-1 DPC) were submitted to the Kansas State University Veterinary Diagnostics Laboratory (KSVDL) for PCR testing of Bovine Respiratory Disease (BRD) Complex pathogens and Influenza D. Samples with Ct values <36 were considered positive; Ct values between 36 and 39 were considered suspect/inconclusive; Ct values >39 were considered negative.

|  | **Turbinate Pool** | | **Trachea/Bronch Pool** | | **Lung Pool** | | **Mandibular lymph node** | |
| --- | --- | --- | --- | --- | --- | --- | --- | --- |
| **Calf ID** | **IDV** | **SARS-CoV-2** | **IDV** | **SARS-CoV-2** | **IDV** | **SARS-CoV-2** | **IDV** | **SARS-CoV-2** |
| **HT1** | 36.61* | 37*,ND | 36.79* | 26,31,33,35 | ND | ND,38*,ND | 38.36* | 34 |
| **HT8** | 38.56* | ND/ND | ND | 26,32,35,ND | ND | 37*,ND,ND | NT | ND |
| **678** | ND | ND/ND | 32.61 | ND | 38.45* | ND | 33.68 | 24 |

**Supplementary Table 2.** **Influenza D virus RNA and SARS-CoV-2 RNA co-detected in tissues at 3 DPC.** At 3 DPC, inactivated RNA obtained from fresh tissues (nasal turbinate, trachea, bronchi, mandibular lymph node and lungs) which were individually tested by RT-qPCR for SARS-CoV-2 were pooled and submitted to the Kansas State Veterinary Diagnostics Laboratory (KSVDL) for RT-qPCR testing of Influenza D virus (IDV). Results for individual tissues (SARS-CoV-2) or results of pooled IDV PCR are displayed as Ct or not detected (ND). Occasionally, both SARS-CoV-2 RNA and IDV RNA were found present in these tissues. NT = Not tested; DPC = Day post challenge; *asterisks (*)* represent suspect positive samples were 50% of the PCR replicates have a Ct.

| **Sample ID** | **Reference name** | **Reference length** | **Mapped reads** | **Minimum coverage** | **Maximum coverage** | **Average coverage** | **Consensus length** | **Fraction of reference covered** |
| --- | --- | --- | --- | --- | --- | --- | --- | --- |
| **Cattle Inoculum** | SARS-CoV-2/hu/CO-BA2-VTMPRSS-P1/2022 | 29750 | 2347403 | 0 | 41680 | 9615.60 | 29657 | 0.997 |
| **HT9 Nasal Swab 2 DPC** | SARS-CoV-2/hu/CO-BA2-VTMPRSS-P1/2022 | 29750 | 8022 | 0 | 407 | 31.85 | 20308 | 0.682 |
| **HT1 Trachea Rostral 3 DPC** | SARS-CoV-2/hu/CO-BA2-VTMPRSS-P1/2022 | 29750 | 1970435 | 0 | 45952 | 8122.40 | 29669 | 0.997 |
| **HT1 Trachea Middle 3 DPC** | SARS-CoV-2/hu/CO-BA2-VTMPRSS-P1/2022 | 29750 | 1514168 | 0 | 44739 | 6263.25 | 29669 | 0.997 |
| **HT1 Trachea Proximal 3 DPC** | SARS-CoV-2/hu/CO-BA2-VTMPRSS-P1/2022 | 29750 | 526001 | 0 | 21943 | 2058.07 | 26064 | 0.876 |
| **HT8 Trachea Rostral 3 DPC** | SARS-CoV-2/hu/CO-BA2-VTMPRSS-P1/2022 | 29750 | 2202222 | 0 | 34558 | 8975.87 | 29669 | 0.997 |
| **HT8 Trachea Middle 3 DPC** | SARS-CoV-2/hu/CO-BA2-VTMPRSS-P1/2022 | 29750 | 903475 | 0 | 29759 | 3700.67 | 29669 | 0.997 |
| **673 Retropharyngeal Lymph Node 7 DPC** | SARS-CoV-2/hu/CO-BA2-VTMPRSS-P1/2022 | 29750 | 72032 | 0 | 3729 | 293.52 | 22894 | 0.769 |

**Supplementary Table 3. Results of next generation sequencing.** Samples were sequenced by next generation sequencing (NGS) using the Illumina NextSeq platform. The mapping data for samples which were included in analysis for strain competition are displayed. Given the similarity in mapping data between SARS-CoV-2/Omicron BA.2 and SARS-CoV-2/Delta, only data for reads mapped to SARS-CoV-2/Omicron BA.2 are shown. Further analysis and variant calling were completed with the VirStrain software package.


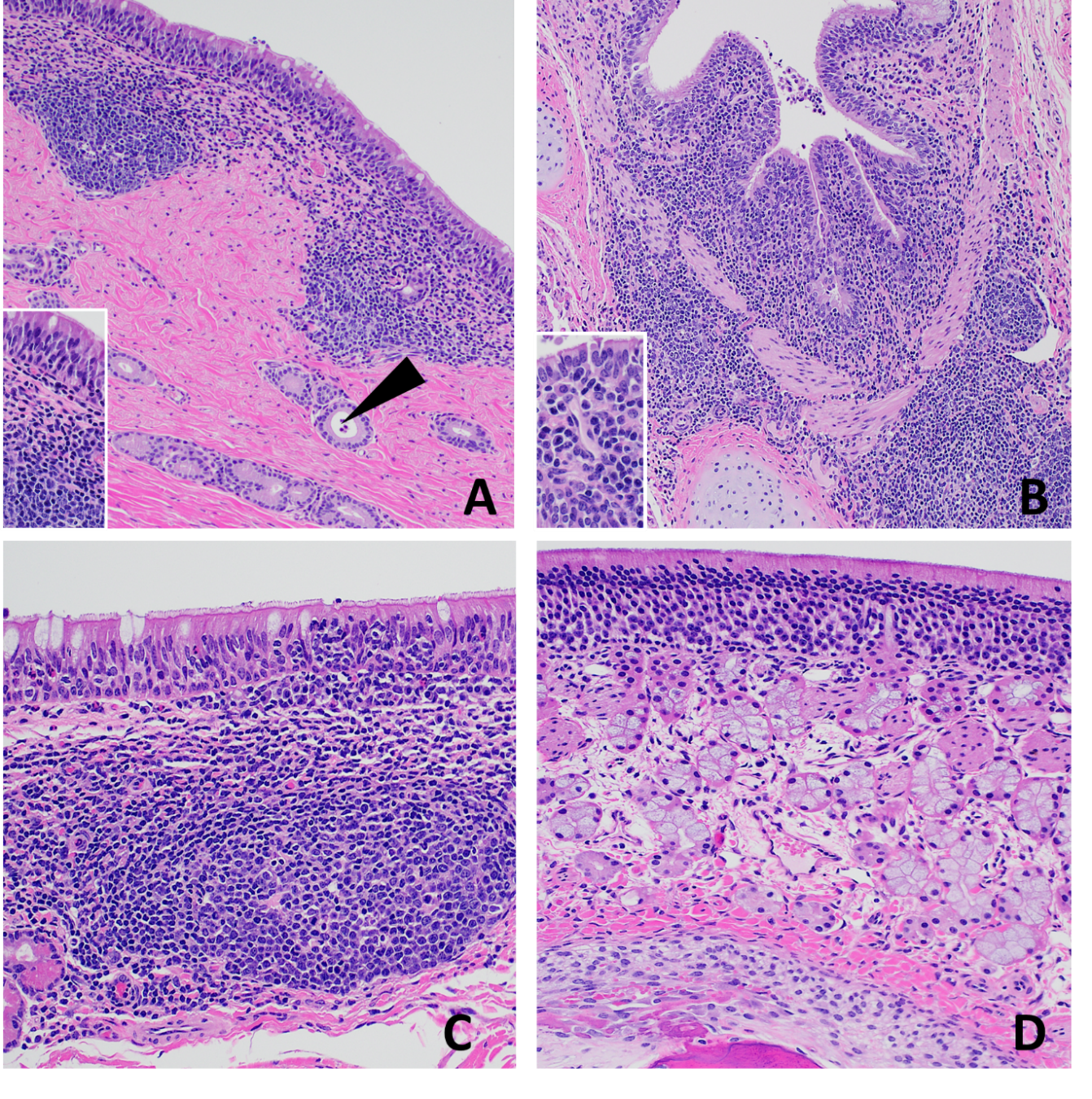


**Supplementary Figure 2**. **Histopathology of 20 DPC respiratory tissues of principal-infected calf 88.** (A) The respiratory epithelium displayed mild lymphocyte and neutrophil transmigration (insert). Mononuclear inflammatory cells expanded the lamina propria overlying dense follicular aggregates of lymphocytes, plasma cells and macrophages. Inflammation (lymphocytes and plasma cells) occurred in and among submucosal glands that sporadically contained degenerate cellular debris (arrowhead). (B) Lymphocytes, plasma cells, macrophages, and lesser numbers of neutrophils segmentally and markedly expanded the mucosa and extended into and among submucosal glands forming large follicular structures. (C) The rostral turbinates had lymphoplasmacytic to neutrophilic infiltrates with epithelial transmigration. Loose and dense sheets of mixed lymphocytic and histiocytic inflammation occurred in the subjacent lamina propria commonly overlying follicular aggregates in the deep submucosa and among glands. (D) The olfactory mucosa of the ethmoturbinates is within normal limits. H&E, 40-400× total magnification.
